# Supplementary material for: Motor cortex gates distractor stimulus encoding in sensory cortex
Source: Nat Commun. 2023 Apr 13;14:2097. doi: 10.1038/s41467-023-37848-4 (PMC10102016; doi:10.1038/s41467-023-37848-4)
Supplement: Supplementary file 1 — Supplementary Information [file 41467_2023_37848_MOESM1_ESM.pdf]

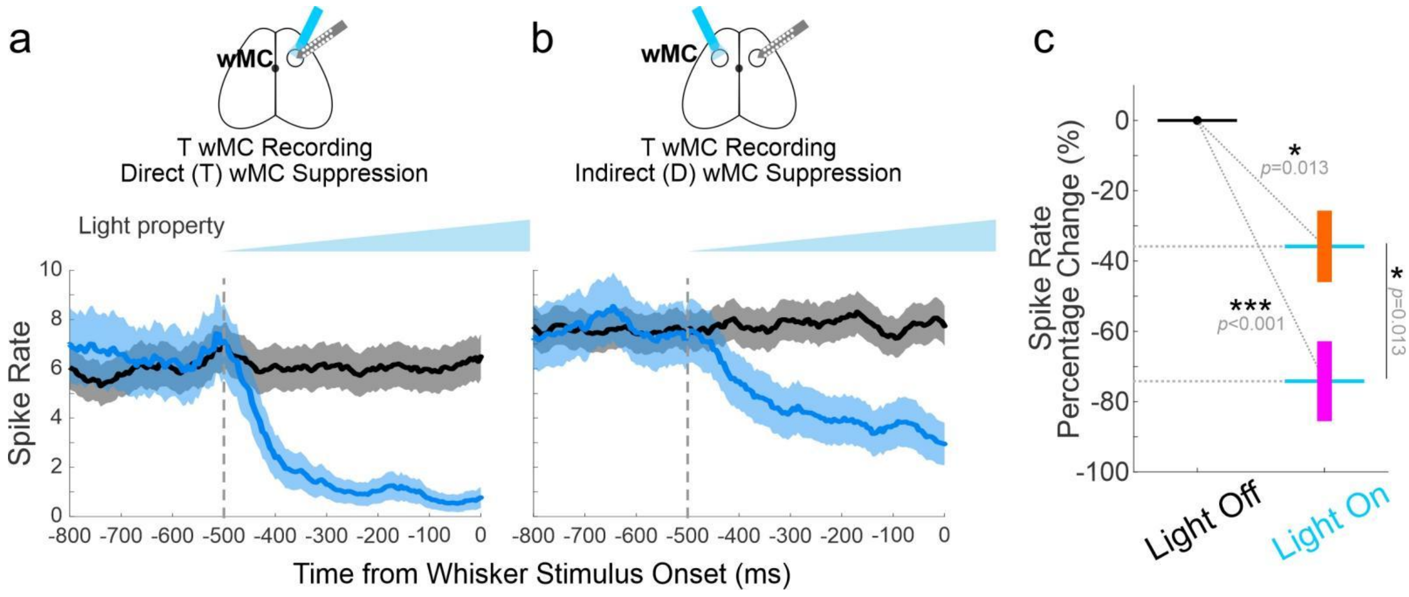

Supplementary Fig.1: Calibration and comparison of wMC suppression efficiency. (a) Neuronal activity of putative excitatory neurons recorded in target-aligned wMC during task performance with and without target-aligned wMC suppression from an example session. Top: Illustration of our method of recording target-aligned wMC neurons with target-aligned wMC suppression (direct). Bottom: neuronal spike rate (averaged across all the trials in one session) for target-aligned wMC suppressed (blue, light on) and control (black, light off) trials. (b) As same as [a], but for distractor-aligned wMC suppression (indirect). Both [a] and [b] are from the same wMC recording session, conducted in block design. (c) Statistical result from all recording sessions, quantifying changes in average spike rates of putative excitatory neurons in a 300ms window before whisker stimulus onset (-300~0 ms period shown in [a] and [b]). Asterisks above each dataset refer to statistical comparisons to control trials, the line connecting the datasets refers to the comparison of direct vs indirect optogenetic suppression. Two-tailed paired t-tests were conducted, without correction for multiple comparisons. Data are represented as mean ± SEM for [a-c].

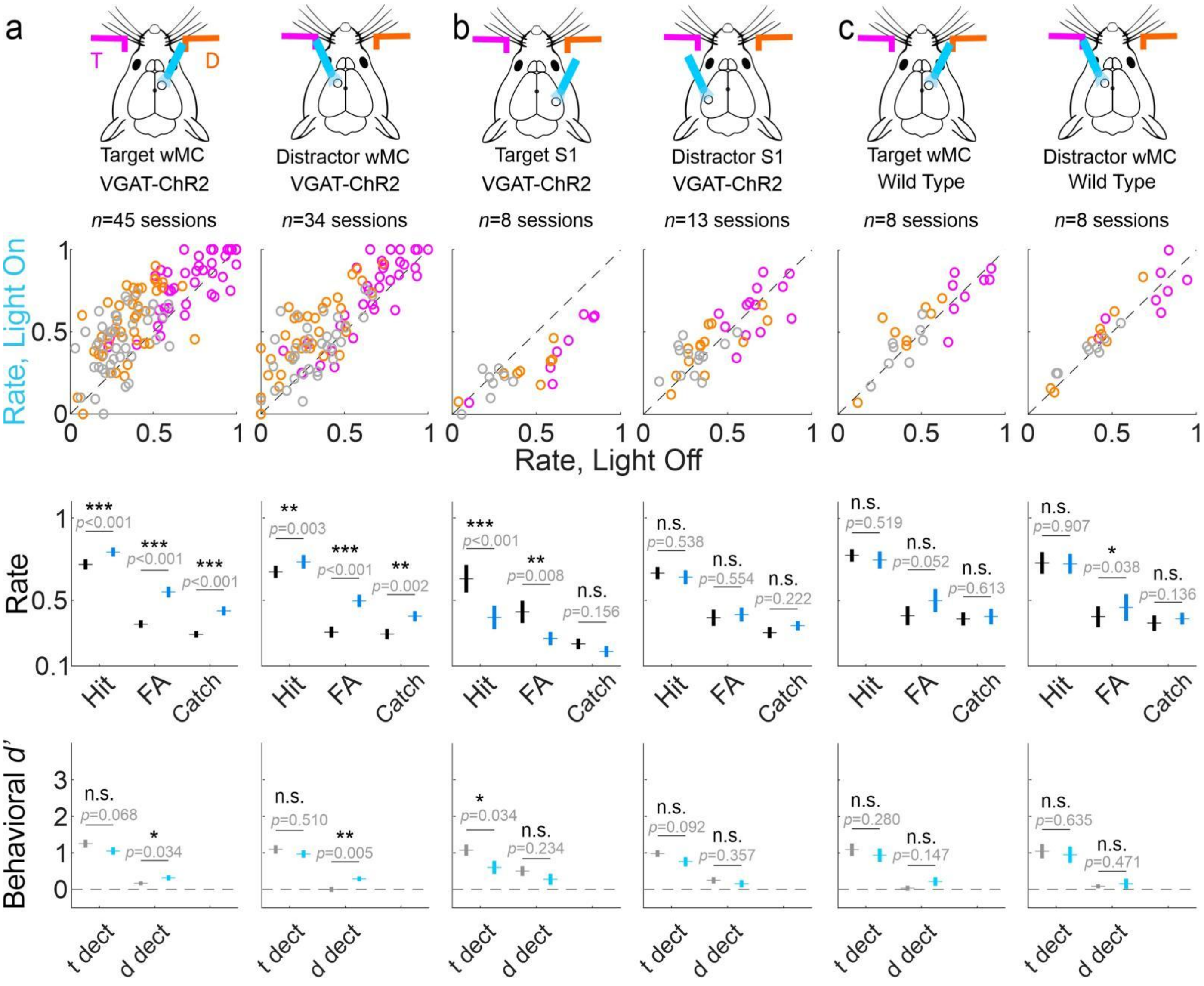

Supplementary Fig. 2: Behavioral effects of optogenetic suppression in VGAT-ChR2 mice and wild type mice for small amplitude whisker stimuli. Presentation is identical to Fig. 1 g-i. Sample sizes are shown in the figure. Two-tailed paired t-tests were conducted, without correction for multiple comparisons. *p* values are shown in the figure.

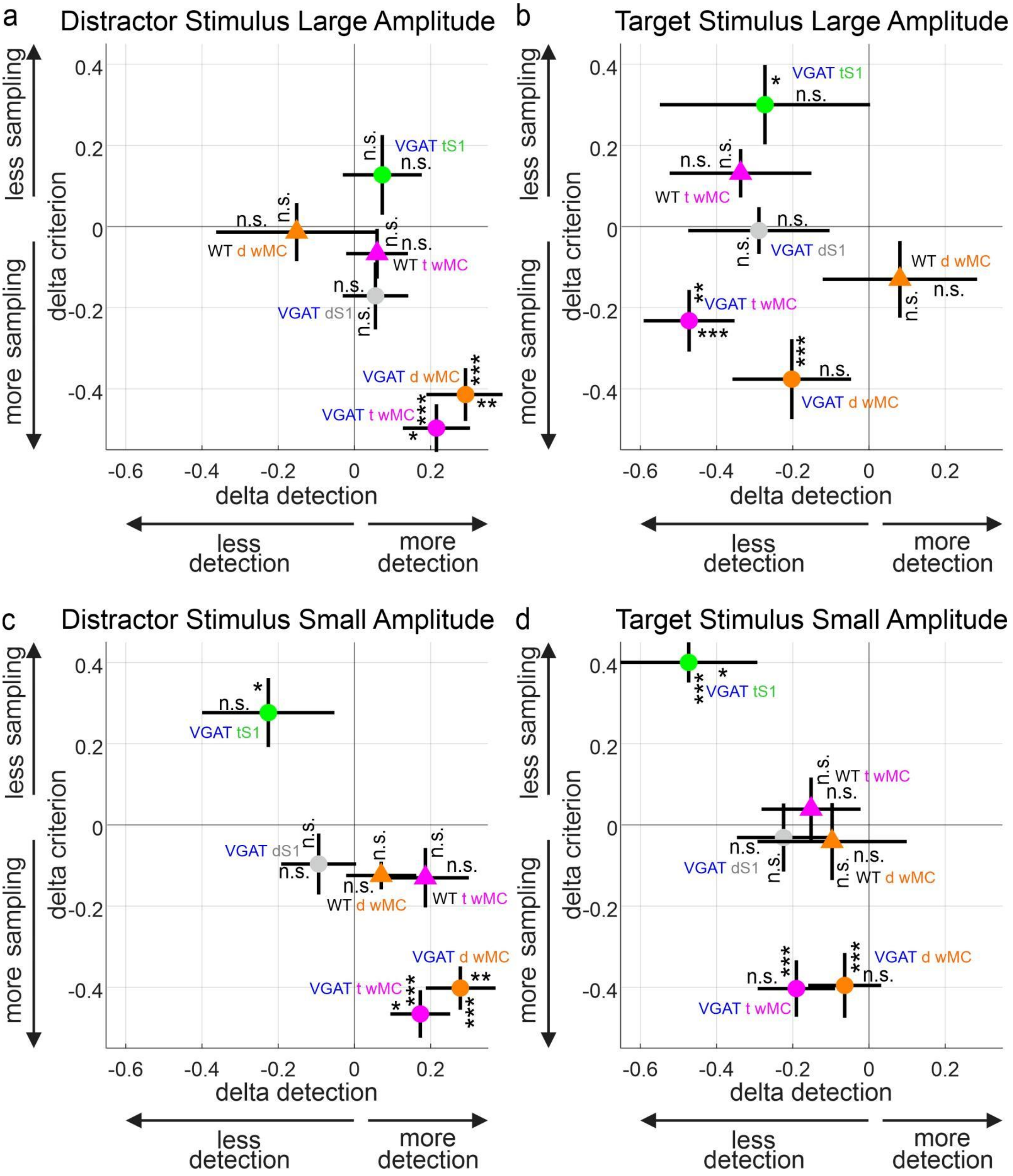

Supplementary Fig. 3: Effects of optogenetic suppression on signal detection theory measures of whisker stimulus detection and criterion. Circles show optogenetic suppression data from VGAT-ChR2 mice: magenta, target-aligned wMC; orange, distractor-aligned wMC; green, target-aligned S1; gray, distractor-aligned S1. Triangles show control data from wild type mice: magenta, target-aligned wMC; orange, distractor-aligned wMC. Scatter plots reflect changes in stimulus detection

(x-axis) vs changes in criterion (tendency to respond, y-axis). Data are plotted for (a) large amplitude distractor whisker stimuli, (b) large amplitude target whisker stimuli, (c) small amplitude distractor whisker stimuli, and (d) small amplitude target whisker stimuli. Sample sizes are the same as Fig. 1 [g-i]. Two-tailed paired t-tests were conducted, without correction for multiple comparisons. Data are represented as mean ± SEM.

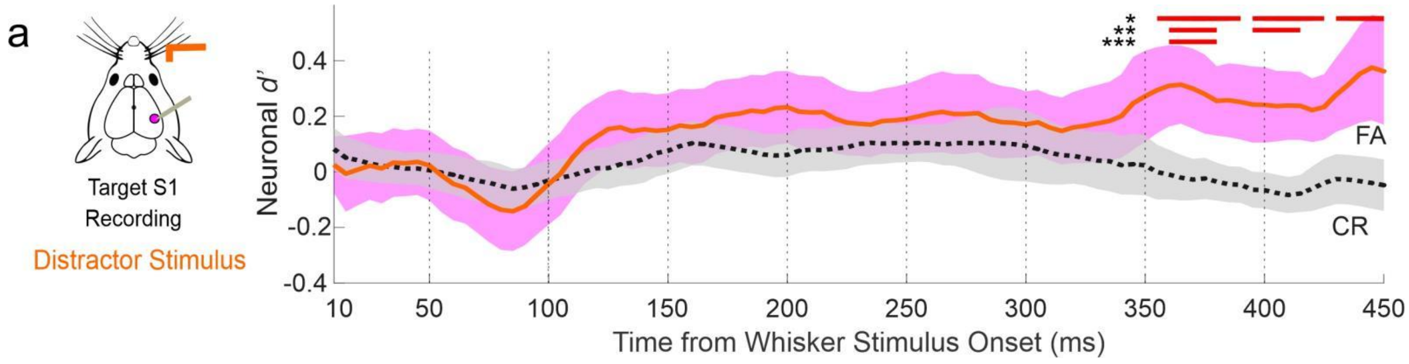

Supplementary Fig. 4: Late increases in target-aligned S1 neuronal activity on false alarm trials. Neuronal *d'* for distractor whisker stimulus responses in target-aligned S1, separated for false alarm (colored) and correct rejection (black) trial outcomes. Same as in Fig. 2 h but extended into the response window (>200 ms post-stimulus). Data are represented as mean ± SEM.

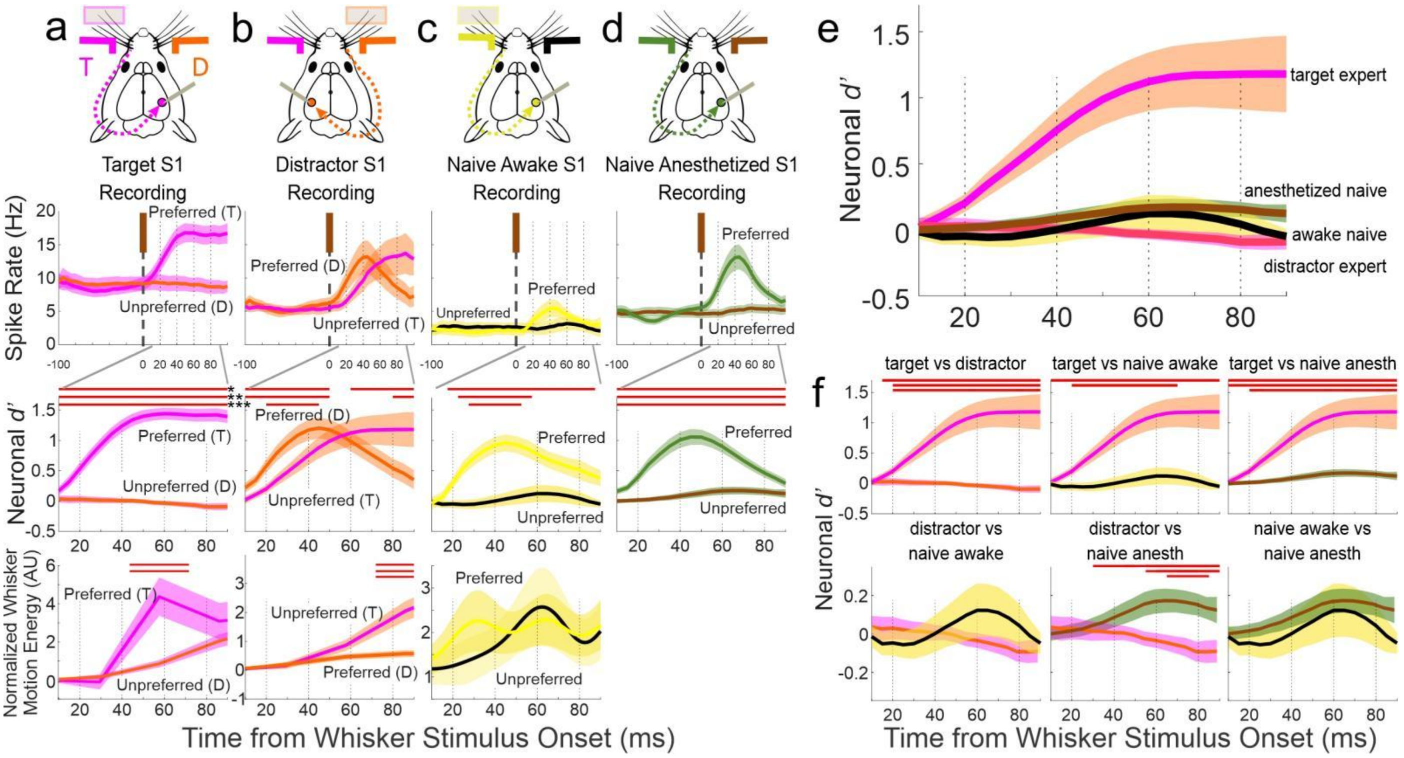

Supplementary Fig. 5: Learning-, state-, and context-dependence of cross-hemispheric (unpreferred) whisker stimulus responses. (a) Recording in target-aligned S1 and target whisker field, comparing responses to preferred (target) and unpreferred (distractor) whisker stimuli in expert mice during task performance. Top: Neuronal spike rate; middle: neuronal *d'*: bottom: whisker motion energy. The red bars indicate the epochs in which neuronal *d'* (for middle row) or whisker motion energy (for bottom row) from two recording groups are significantly different from each other; same indication for [b,c,d,f]. Differences in spike rates were not compared for statistical significance. (b) As same as [a] but recording in distractor-aligned S1 or distractor whisker field, comparing responses to preferred (distractor) and unpreferred (target) whisker stimuli in expert mice during task performance. (c) As same as [a] but recording in naive awake mice. (d) As same as [a] but recorded in naive mice under anesthesia. Data are grand averages, combined from all recording sessions. (e) Overlay of neuronal *d'* for unpreferred stimulus encoding; “target” refers to target stimuli in distractor-aligned S1, “distractor” refers to distractor stimuli in target-aligned S1. (f) Pairwise comparison of neuronal *d'* for unpreferred stimulus encoding from [e]. Data are represented as mean ± SEM.

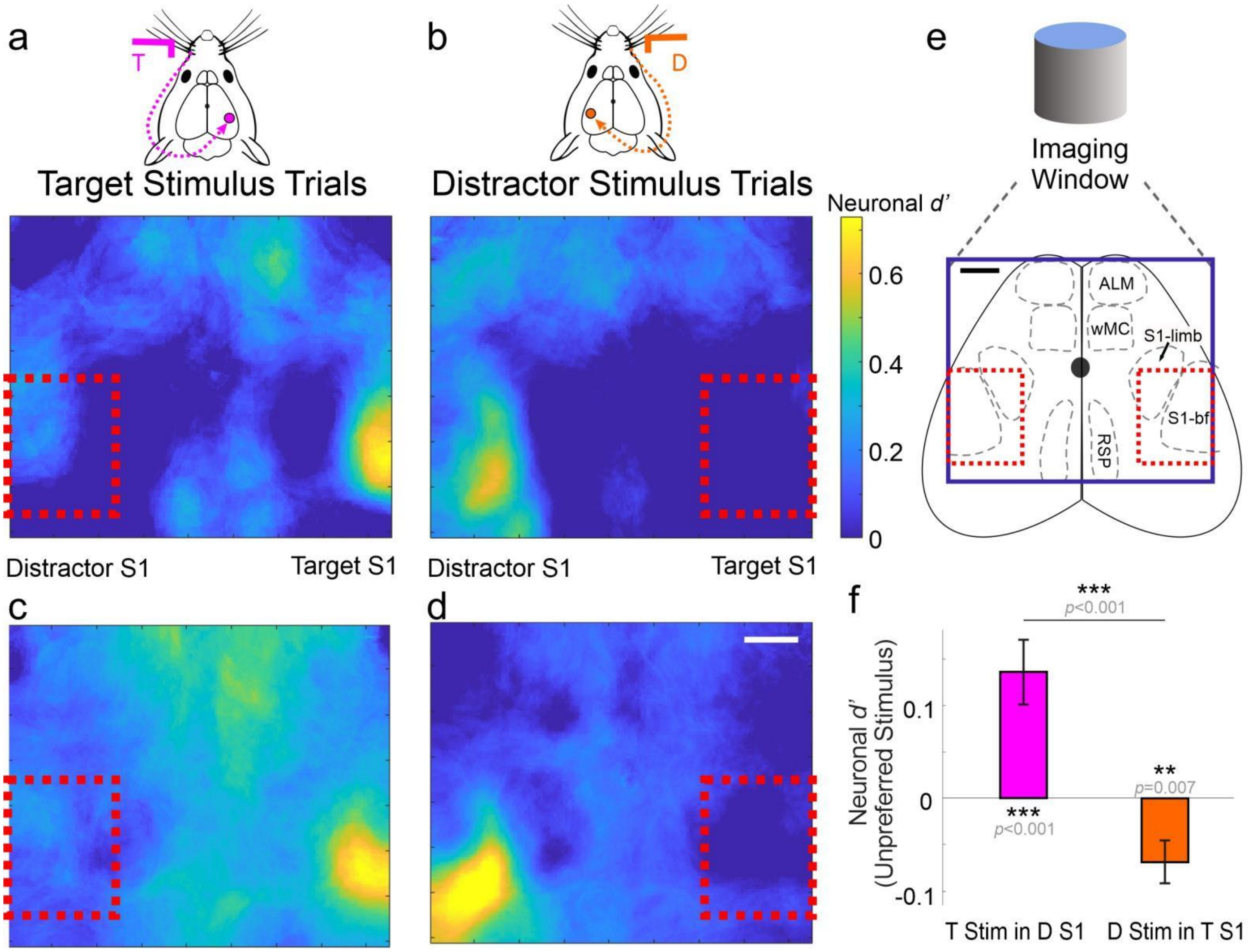

Supplementary Fig. 6: Widefield GCaMP6 imaging evidence for asymmetric activation of target-aligned and distractor-aligned S1 in response to their unpreferred whisker stimuli. (e) A reference brain map, showing the imaging window presented in [a-d], with a few task-relevant regions indicated. ALM: anterior lateral motor cortex; S1-limb: primary somatosensory cortex, limb regions; S1-bf: primary somatosensory cortex, barrel field; RSP: retrosplenial cortex. The analysis regions of interest (ROIs) are outlined in red. (a-d) Data from two example imaging sessions. Stimulus encoding (neuronal *d'*) was computed for each pixel as the separation between whisker stimulus absent and stimulus present, for target whisker stimulus trials [a,c] and distractor whisker stimulus trials [b,d]. Activations evoked by unpreferred whisker stimuli were quantified (target whisker stimuli in distractor-aligned S1 and distractor whisker stimuli in target-aligned S1). (f) Quantification of averaged whisker stimulus encoding (neuronal *d'*) in the selected ROIs calculated across n=40 sessions. Above each bar are results from two-tailed one-sample t-tests to determine

differences from zero within each ROI, and the line between bars reflects results from a two-tailed paired t-tests to determine differences between ROIs. Data are represented as mean ± SEM. Corrections for multiple comparisons were not performed. T stim in D S1: target whisker stimulus encoding in distractor-aligned S1; D stim in T S1: distractor whisker stimulus encoding in target-aligned S1. Scale bars in [e, d]: 1 mm.

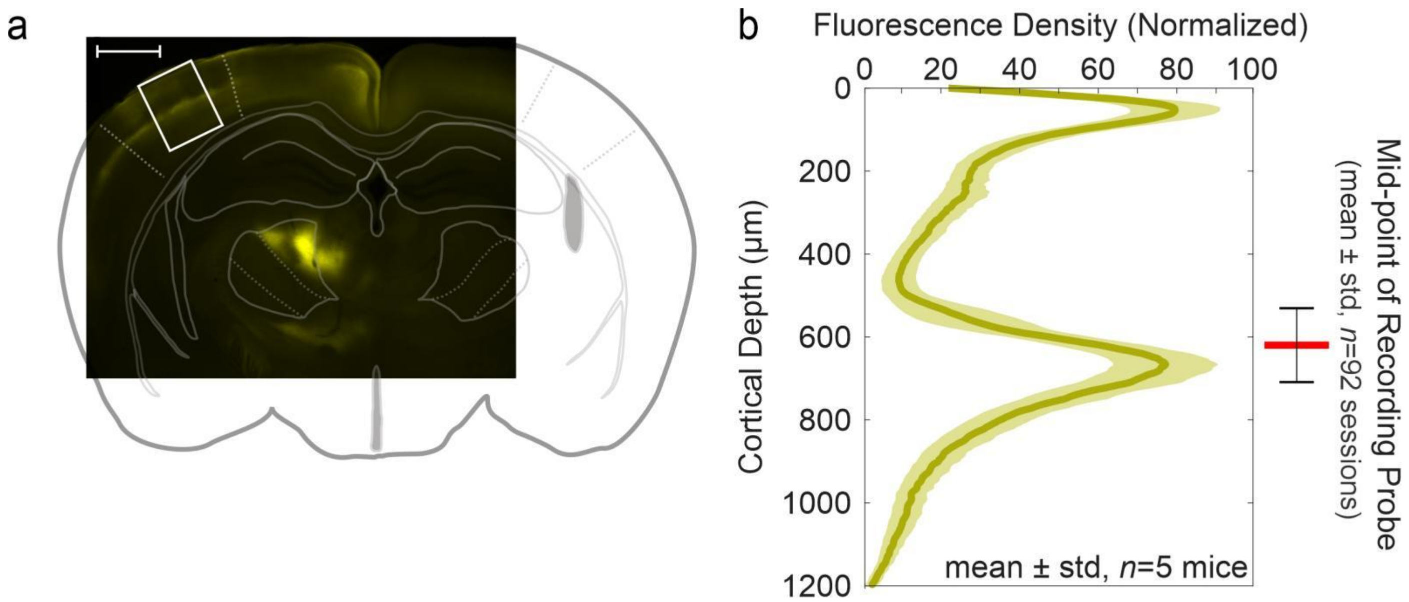

Supplementary Fig. 7: Laminar profile of the wMC→S1 direct feedback pathway. We performed viral-mediated labeling of wMC axons and terminals in S1. (a) An example histological section showing YFP fluorescence in a coronal brain section through S1, overlayed by a schematic reference of brain structure. Dotted lines indicate the boundary of the S1 barrel field, the solid rectangle indicates the ROI used for laminar expression analyses. Scale bar, 1000 µm. (b) Quantification of fluorescence intensity from wMC→S1 projections from 5 mice, demonstrating high axonal densities in layers 1 and 5 and sparing layer 4. On the right, the red line and error bars indicate the mid-point of the recording probes from all S1 recording sessions.

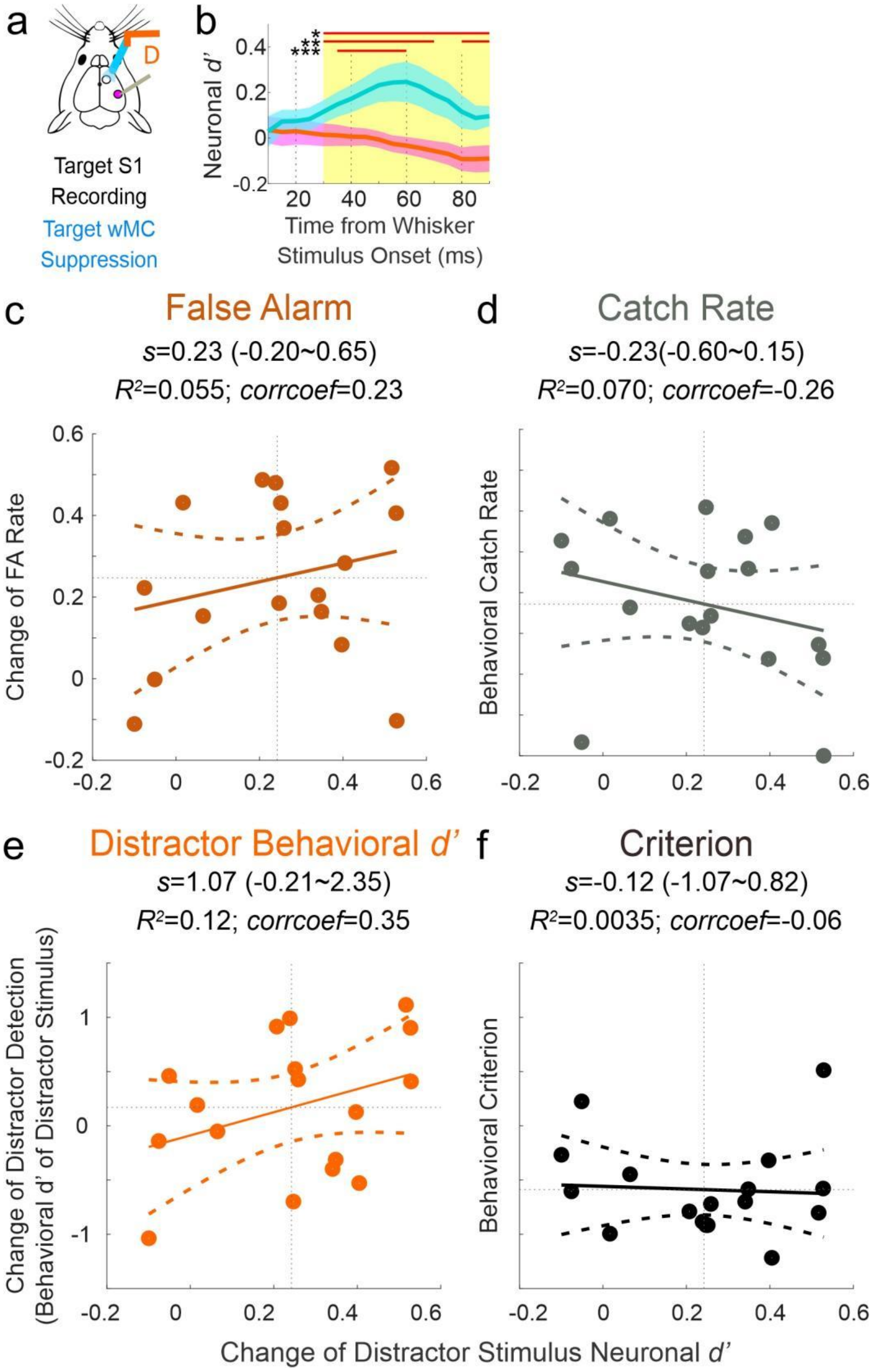

Supplementary Fig. 8: Increased distractor stimulus encoding in target-aligned S1 correlates with increased behavioral distractor detection. (a) Illustration of recording distractor whisker stimulus responses in target-aligned S1 while optogenetically suppress target-aligned wMC. (b) Calculation of the change of distractor stimulus encoding, as the average neuronal *d'* of light-on trials minus the average neuronal *d'* of light-off trials across 30ms~100ms after distractor stimulus onset (indicated by the yellow shade). Data are represented as mean ± SEM. (c) Scatter plot and linear regression comparing the change of distractor stimulus neuronal *d'* (x-axis) and the change of

false alarm rate (y-axis). Each data point is one recording-behavioral session (*n*=17 sessions). Solid colored line indicates the linear fit and the dashed colored lines indicate the 95% confidence interval. Dashed gray lines indicate the averaged values of data along x and y axes. (d) As same as [c] but the y-axis is the change of catch rate. [e] As same as [c] but the y-axis is the change of distractor detection (behavioral *d'* of the distractor stimulus). (f) As same as [c] but the y-axis is the change of distractor criterion.

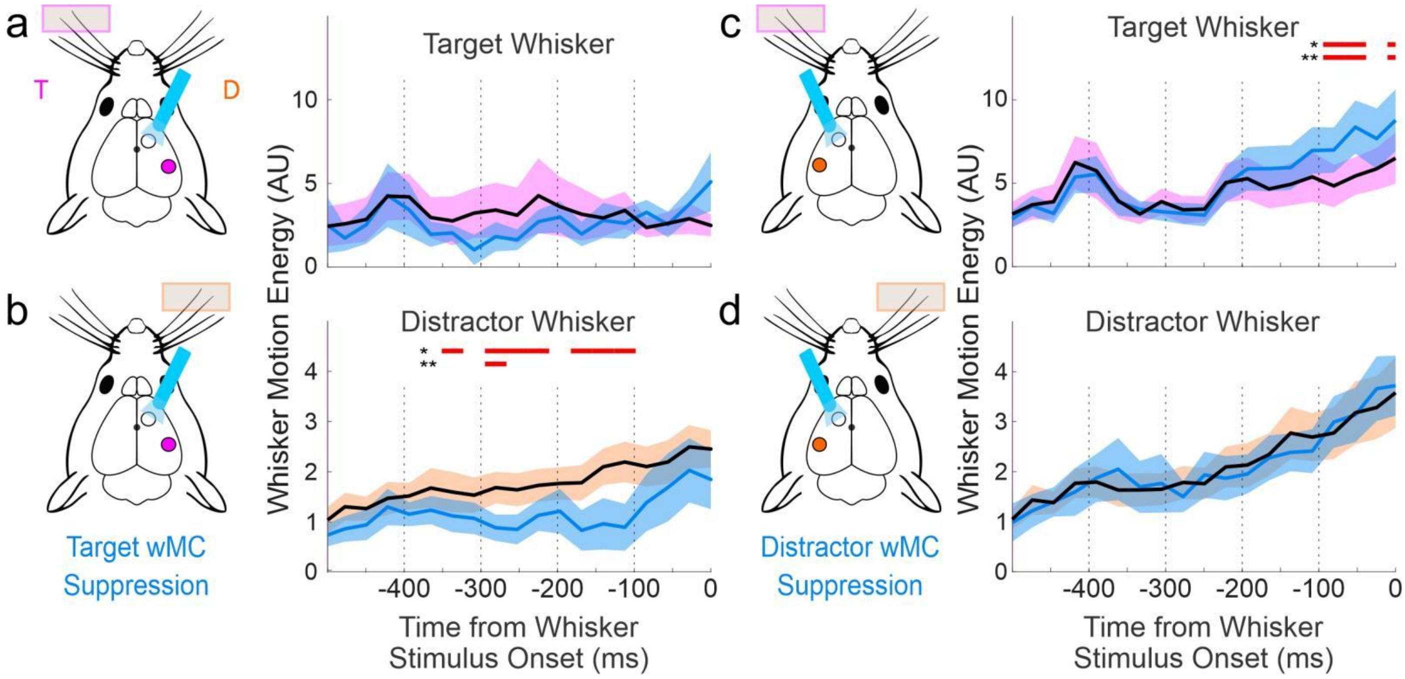

Supplementary Fig. 9: Optogenetic suppression of wMC does not systematically change whisker motion energy. (a) Left: Illustration of simultaneous optogenetic suppression of target-aligned wMC and whisker imaging of the target whisker field. Right: Whisker motion energy of the target whisker field during control trials (light-off, magenta) and wMC suppression trials (light-on, blue) before whisker stimulus onset. (b) As same as [a] but for distractor whisker fields. (c,d) As same as [a,b], but for distractor-aligned wMC suppression. Data are represented as mean ± SEM.

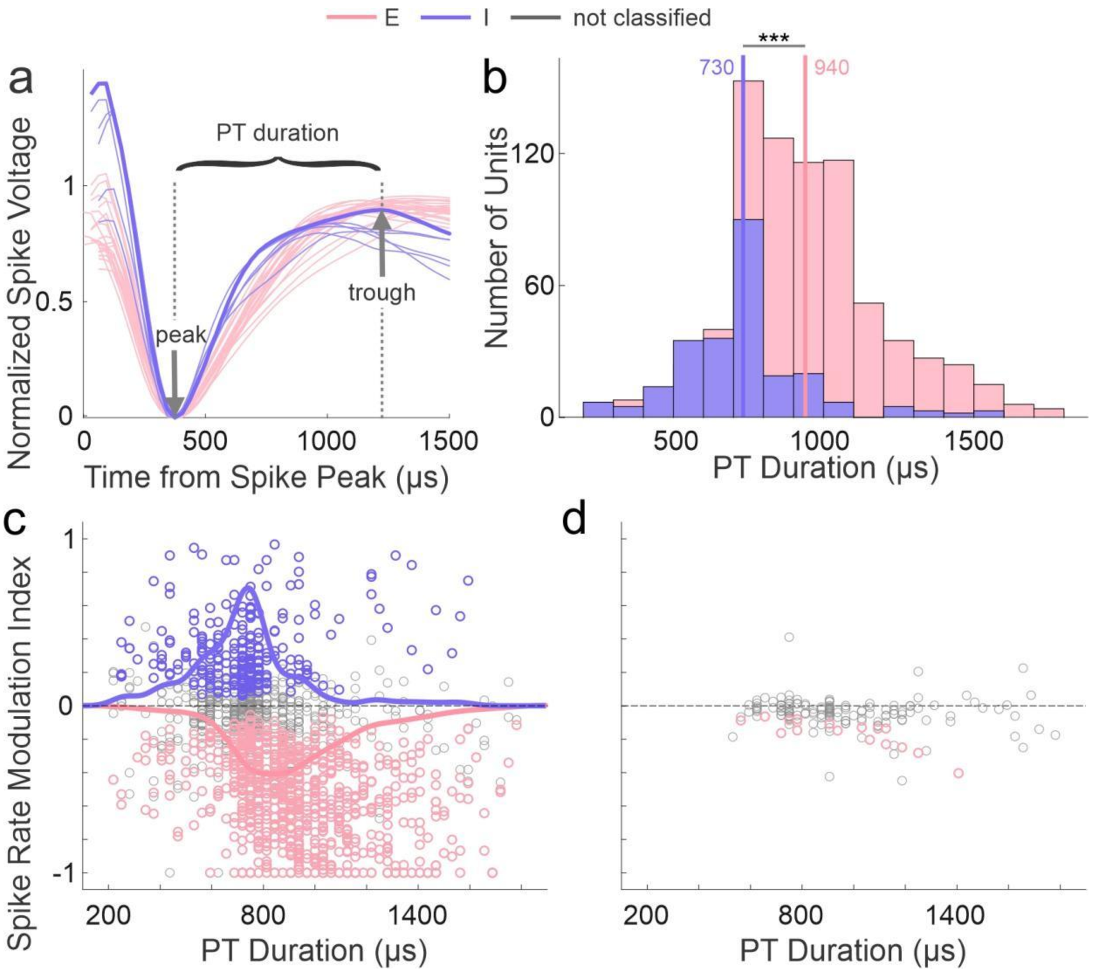

Supplementary Fig. 10: Opto-tagging method and verification. Putative excitatory or putative inhibitory neurons are assigned based on significant decreases or increases, respectively, in spike rate during opto-stimulation within the S1 recording site, using $p<0.1$ as the significance threshold. (a) Spike widths of putative inhibitory (purple) and excitatory (pink) neurons from one example session. Spike width is calculated as peak-trough (PT) duration. (b) Histogram of putative excitatory and inhibitory neuron counts of different spike widths. Two vertical lines indicate the average spike widths of each population, which are significantly different from each other (two-tailed two sample t-test, $p=1.73\times10^{-28}$). Putative inhibitory neurons have shorter spike widths, as expected, yet there is substantial overlap between these populations. (c) Distribution of spike width versus opto-tagging modulation index from VGAT-ChR2 mice (n=72 recording sessions). Each data point is one unit, and the two curves show the density distributions. Modulation index (MI) is calculated as the change in spike rate during opto-tagging. MI>0 indicates units enhanced by direct light stimulation; MI<0 indicates units suppressed by direct light stimulation. 1613

neurons are recorded, in which 762 neurons (47.24%) are characterized as putative excitatory neurons, 246 neurons (15.25%) are characterized as putative inhibitory neurons, 605 neurons (37.51%) are not significantly modulated by opto-tagging and therefore unassigned. (d) As same as [c] but the units were recorded from control wild type mice (*n*=6 recording sessions). In wild type mice, 155 neurons were recorded, in which 16 neurons (10.03%) are significantly modulated by opto-tagging, consistent with an expected false positive rate of 10%.

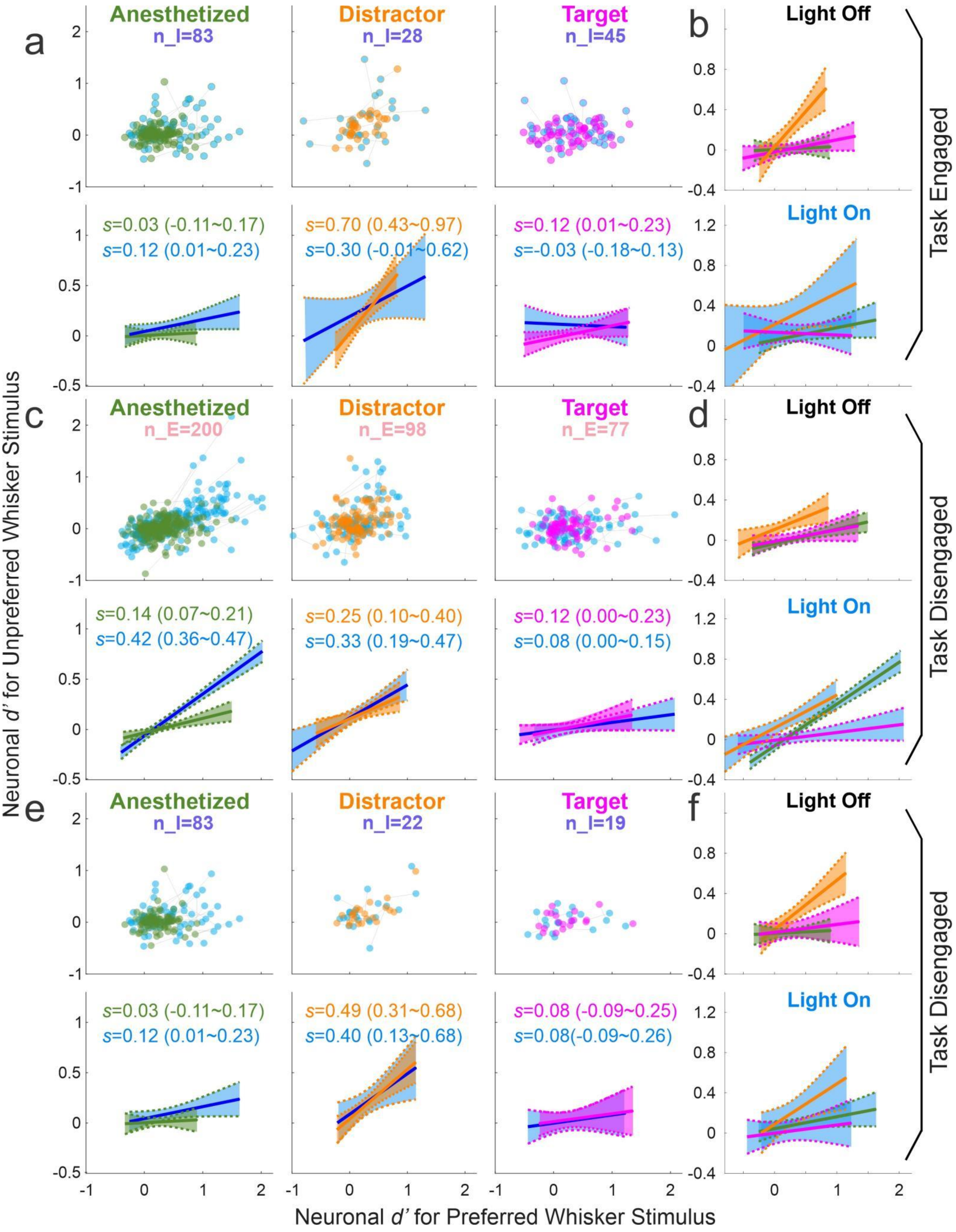

Supplementary Fig. 11: S1 neuronal encoding (neuronal *d'*) of preferred and unpreferred whisker stimuli, according to cell type and task engagement. (a) As same as Fig. 4 d-f, but for putative

inhibitory neurons recorded: under anesthesia (1st column), in distractor-aligned S1 in awake behaving mice (2nd column), and in target-aligned S1 in awake behaving mice (3rd column). (b) The overlay of the control (top) and wMC suppression (bottom) linear fits from the second row of [a]. (c,d) As same as Fig. 4 d-g (putative excitatory neurons), but from disengaged awake recording sessions. (e,f) As same as Fig. 4 d-g, but for inhibitory neurons recorded from disengaged awake recording sessions. Anesthetized data is duplicated for “engaged” and “disengaged” categories and included for reference. Note, for putative excitatory neurons when task disengaged [c], the whisker stimulus selectivity profiles for distractor-aligned (orange) and target-aligned (magenta) S1 neurons are not significantly different. Furthermore, for these conditions, wMC suppression (blue) does not change stimulus selectivity. These data are in marked contrast to S1 excitatory neurons during task engagement (Fig. 4 e,f).

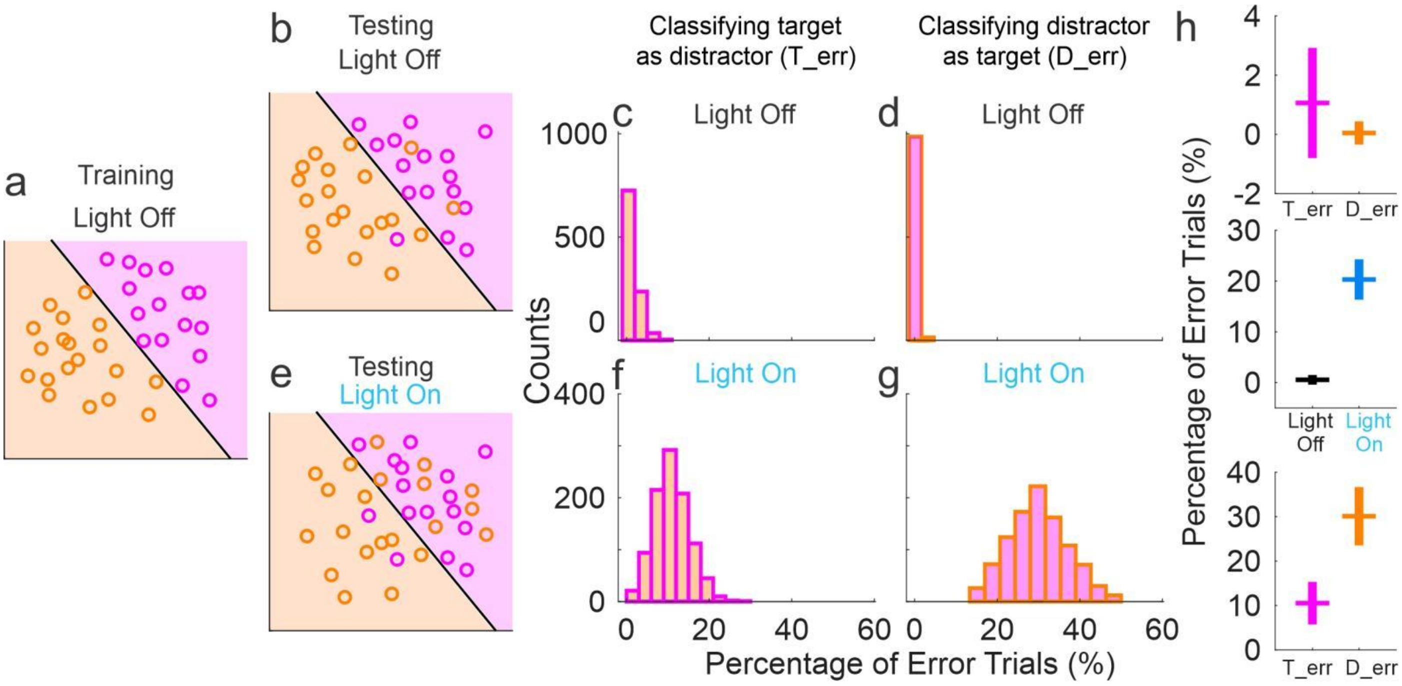

Supplementary Fig. 12: Classification outcomes from linear discriminant analyses of trial types. (a) Illustration of a hypothetical linear model separating trial types based on training data. Each circle indicates one trial. Magenta circles indicate true target trials; orange circles indicate true distractor trials. The line indicates the classifier. In this example, the model correctly classifies all target and distractor trials. (b) Illustration of classification of hold-out control trials. (c,d) Classification data from hold-out control trials. Histograms of percent error bins for miss (c, classifying target as distractor) and false alarm (d, classifying distractor as target) errors, from 1000 iterations. (e) Illustration of classification of wMC suppression trials. (f,g) Classification data from light-on wMC suppression trials, plotted as in [c,d]. (h) Mean ± standard deviation error rate (err) comparisons for [c] vs [d] (top,T_err %: 1.06±1.86; D_err %: 0.05±0.39), [f] vs [g] (bottom, T_err %: 10.52±4.79; D_err %: 30.09±6.56), and the average of [c] and [d] vs the average of [f] and [g] (middle, Light-off %: 0.55±0.95, Light-on %: 20.31±3.98).

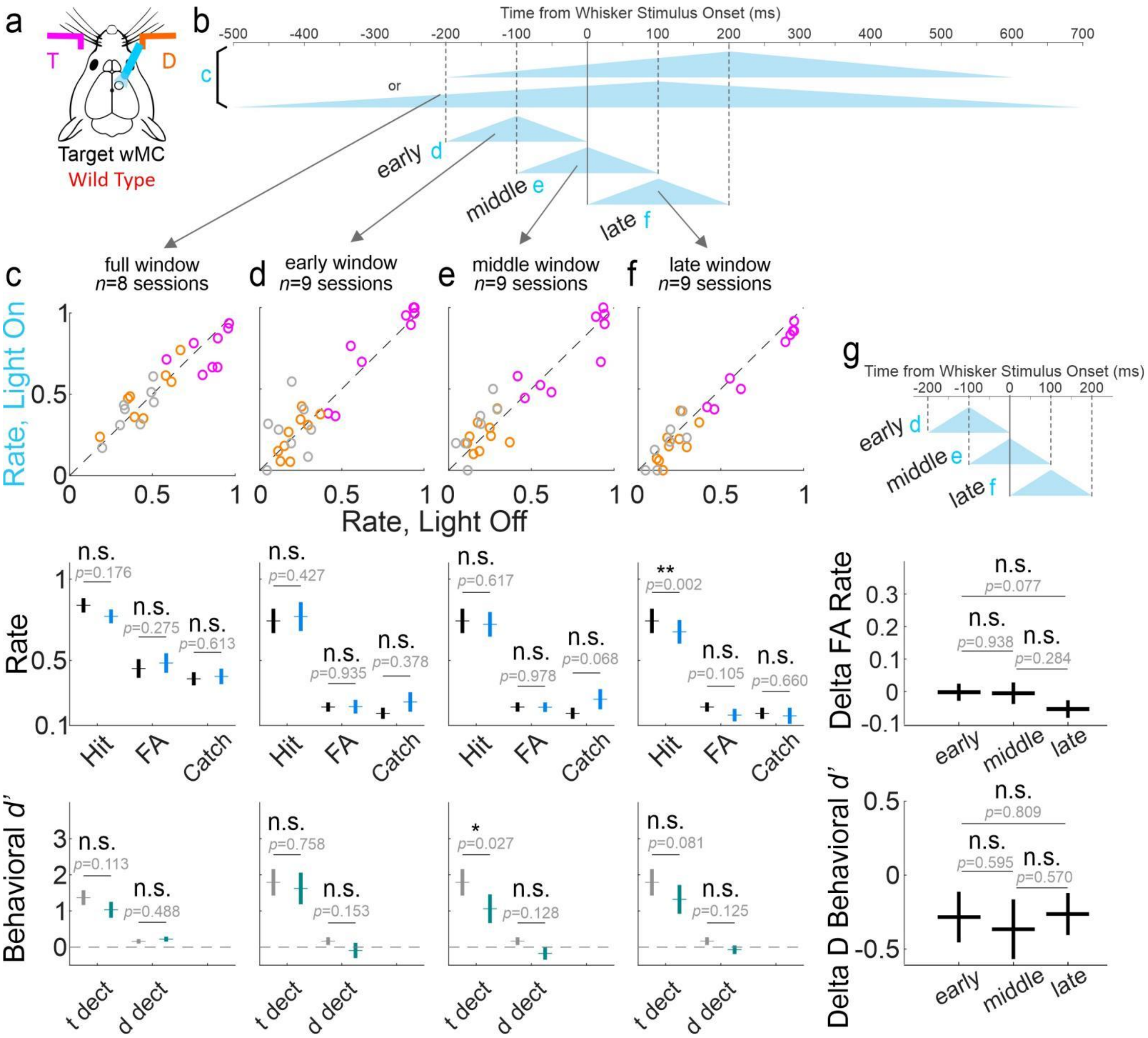

Supplementary Fig. 13: Behavioral effects from wMC suppression are not due to artifacts of opto-stimulation. As same as Fig. 6 but for wild type control mice.

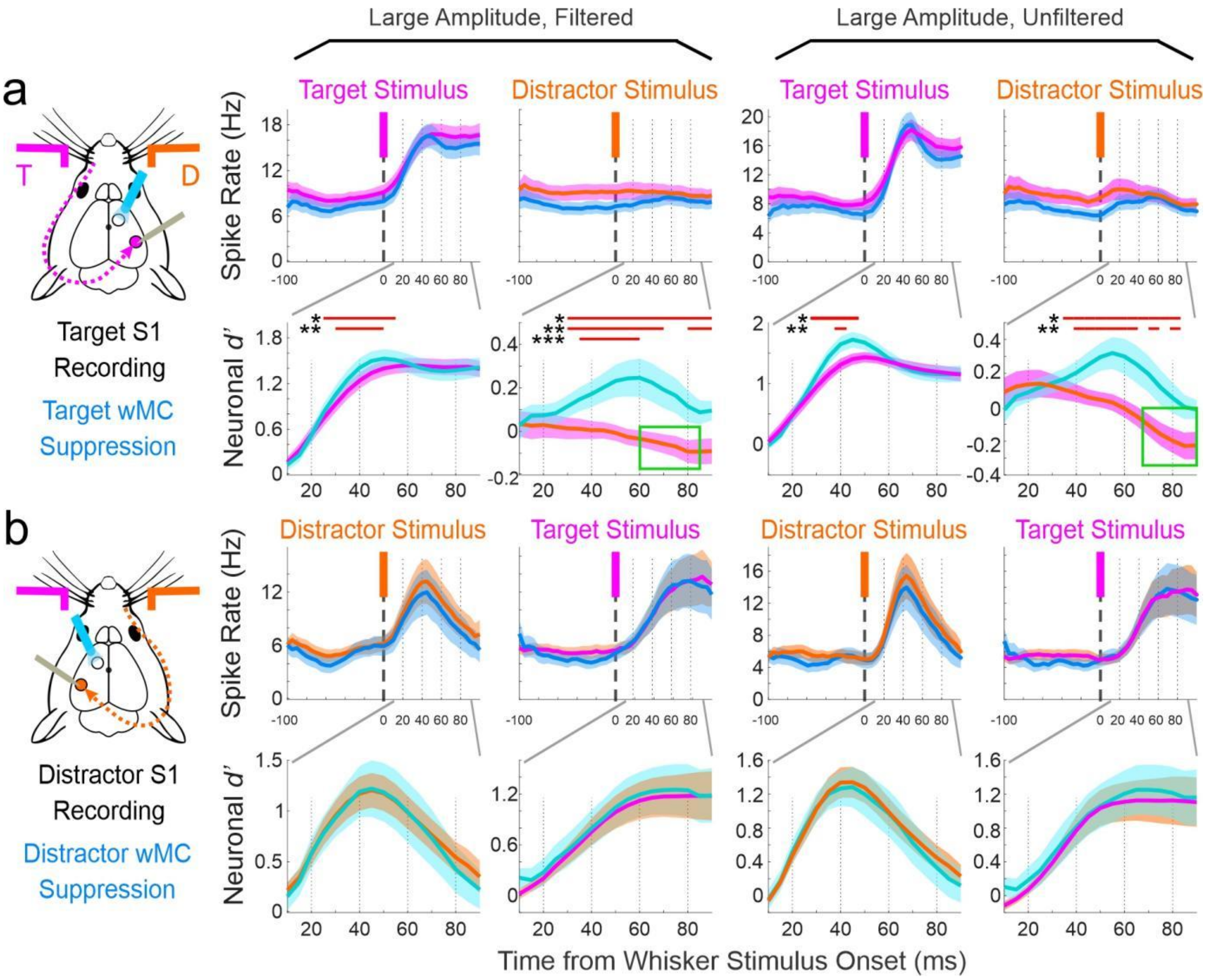

Supplementary Fig. 14: Comparison of neuronal spike rates and neuronal *d'* of S1 neurons during wMC suppression for 10-20 Hz filtered and unfiltered data (see "Data inclusion criteria and quality control" in Methods). Organization as presented in Fig. 3 a-f. Data are represented as mean ± SEM.

mouse name	genotype	sex	# of units in total	WDB non-opto	WDB long opto	WDB short opto	WIB	Whisker imaging	awake recording in S1	awake recording in wMC	anesthesia recording	opto tagging
R069	Vgat	F	125		5				5			5
					12							
R070	Vgat	F	406		18				18			18
					13							
R071	Vgat	M			10							
R072	Vgat	M	83								4	4
R075	Vgat	M	254		10				10			10
					6							
R076	Vgat	M	67								3	3
R077	Vgat	F	24								1	1
R078	Vgat	M	195								12	12
R079	Vgat	M	337						13			13
R080	Vgat	M	127								6	6
R081	WT	M	51								2	2
R082	WT	F	104								4	4
R089	Vgat	F	221		8			8				
					6					6		6
						6						
R090	Vgat	F	259		8			8				
					8					8		8
						6						
R099	WT	M			6			6				
				1				1				
					4							
						3						
R100	WT	F			6							
						3						
R101	WT	F				3						
R102	Vgat	F	226				1	1	1			
							6		6			
							1					
R103	WT	F	151				4	4	4			
							3		3			
							1					

Supplementary Table 1: Summary of all the mice used in this study (except for the 5 mice used for histology). Mouse identifications that are coded with the same color indicate littermates. For genotype, “Vgat” indicates VGAT-ChR2 transgenic genotype; “WT” indicates wild type. “# of units in total” indicates the total number of single units recorded from each mouse throughout all sessions. In the remaining columns, numbers indicate the number of behavior and/or recording sessions. “WDB” refers to whisker dependent behavior, as in the selective whisker detection task. “WIB” refers to whisker independent behavior, for habituated behavior not requiring whisker detection. “non-opto” indicates that there were no light-on trials in the session. “long opto” indicates the standard light duration used in most of our optogenetic suppression studies (all optogenetic suppression data except for Fig. 6 and Supplementary Fig. 13) and the “short opto” indicates the light duration used in Fig. 6 d-f and Supplementary Fig. 13 d-f. Red arrows link the same sessions with simultaneous operations under different categories. For example, the first row means for mouse R069, we obtained data from 5 sessions with optogenetic suppression when mice were performing the whisker selective detection task, with simultaneous neuronal recordings in S1 and opto-tagging. We also obtained another 12 sessions from this mouse, but only for

optogenetic suppression when mice were performing the whisker selective detection task (behavior only).

a. Vgat-ChR2 engaged									
		tM1 light: 45 sessions		dM1 light: 34 sessions		tS1 light: 8 sessions		dS1 light: 13 sessions	
		Total trials	out	Total trials	out	Total trials	out	Total trials	out
Large target	Light off	26 ± 14	0	35 ± 15	0	33 ± 12	0	30 ± 1	0
	Light on	11 ± 74	6	14 ± 9	4	17 ± 5	0	14 ± 5	1
Small target	Light off	36 ± 15	0	43 ± 22	0	50 ± 12	0	48 ± 17	0
	Light on	16 ± 7	1	18 ± 10	1	26 ± 9	0	22 ± 7	0
Large distractor	Light off	50 ± 25	0	56 ± 34	0	56 ± 21	0	61 ± 21	0
	Light on	22 ± 11	0	26 ± 18	0	32 ± 13	0	28 ± 10	0
Small distractor	Light off	50 ± 25	0	56 ± 35	0	62 ± 22	0	61 ± 14	0
	Light on	23 ± 1	0	26 ± 15	0	32 ± 11	0	28 ± 8	0
Catch	Light off	38 ± 22	0	42 ± 28	0	62 ± 22	0	56 ± 15	0
	Light on	18 ± 10	1	22 ± 15	2	30 ± 12	0	24 ± 9	0

b. Vgat-ChR2 disengaged					
		tM1 light: 3 sessions		dM1 light: 6 sessions	
		Total trials	out	Total trials	out
Large target	Light off	57 ± 12	0	53 ± 8	0
	Light on	24 ± 12	0	29 ± 8	0
Small target	Light off	58 ± 14	0	58 ± 14	0
	Light on	34 ± 7	0	30 ± 4	0
Large distractor	Light off	32 ± 3	0	31 ± 9	0
	Light on	20 ± 8	0	16 ± 55	0
Small distractor	Light off	36 ± 17	0	36 ± 6	0
	Light on	22 ± 6	0	17 ± 5	0
Catch	Light off	22 ± 5	0	21 ± 4	0
	Light on	13 ± 5	0	13 ± 2	0

c. Wild Type engaged					
		tM1 light: 8 sessions		dM1 light: 8 sessions	
		Total trials	out	Total trials	out
Large target	Light off	65 ± 21	0	67 ± 16	0
	Light on	32 ± 14	0	32 ± 9	0
Small target	Light off	60 ± 18	0	66 ± 12	0
	Light on	31 ± 13	0	29 ± 9	0
Large distractor	Light off	97 ± 27	0	92 ± 21	0
	Light on	47 ± 14	0	44 ± 10	0
Small distractor	Light off	91 ± 20	0	89 ± 15	0
	Light on	43 ± 15	0	43 ± 8	0
Catch	Light off	74 ± 15	0	71 ± 15	0
	Light on	39 ± 9	0	32 ± 12	0

d. Vgat-ChR2 engaged moving light short window: 12 sessions (different trials are interleaved)									
		Light off: 12 sessions		tM1 early light on		tM1 middle light on		tM1 late light on	
		Total trials	out	Total trials	out	Total trials	out	Total trials	out
Target		119 ± 39	0	20 ± 9	0	18 ± 7	0	20 ± 11	0
Distractor		114 ± 44	0	17 ± 6	0	20 ± 6	0	20 ± 6	0
Catch		41 ± 15	0	7 ± 3	4	6 ± 3	3	8 ± 3	1

e. Wild Type engaged moving light short window: 9 sessions (different trials are interleaved)								
	Light off		tM1 early light on		tM1 middle light on		tM1 late light on	
	Total session: 9	out	Total trials	out	Total trials	out	Total trials	out
Target	126 ± 28	0	22 ± 6	0	21 ± 5	0	20 ± 4	0
Distractor	139 ± 39	0	22 ± 7	0	26 ± 11	0	25 ± 11	0
Catch	52 ± 15	0	9 ± 4	0	6 ± 3	2	8 ± 4	2

Supplementary Table 2: Summary of session and trials numbers from the selective whisker detection task with opto-stimulation used in this study. (a,d) Data from all sessions of VGAT-ChR2 transgenic mice during task engagement. (b) Data from all sessions of VGAT-ChR2 transgenic mice during task disengagement. (c,e) Data from all sessions of wild type mice during task engagement. “Out” indicates number of sessions that were excluded for any calculation due to insufficient trial numbers (see “Data inclusion criteria and quality control:” in “Methods). For (a-c) the standard (“long opto”) opto-stimulation profiles were used, for (d-e) the transient, varying (“short opto”) opto-stimulation profiles were used.
